# Supplementary material for: History of primary-series and booster vaccination and protection against Omicron reinfection
Source: Sci Adv. 2023 Oct 4;9(40):eadh0761. doi: 10.1126/sciadv.adh0761 (PMC10550237; doi:10.1126/sciadv.adh0761)
Supplement: Supplementary file 1 — Figs. S1 to S5 Tables S1 to S3 [file sciadv.adh0761_sm.pdf]

Science Advances
AAAS

Supplementary Materials for

History of primary-series and booster vaccination and protection against Omicron reinfection

Hiam Chemaitelly *et al.*

Corresponding author: Hiam Chemaitelly, hsc2001@qatar-med.cornell.edu;

Laith J. Abu-Raddad, lja2002@qatar-med.cornell.edu

Sci. Adv. **9**, eadh0761 (2023)
DOI: 10.1126/sciadv.adh0761

This PDF file includes:

Figs. S1 to S5
Tables S1 to S3

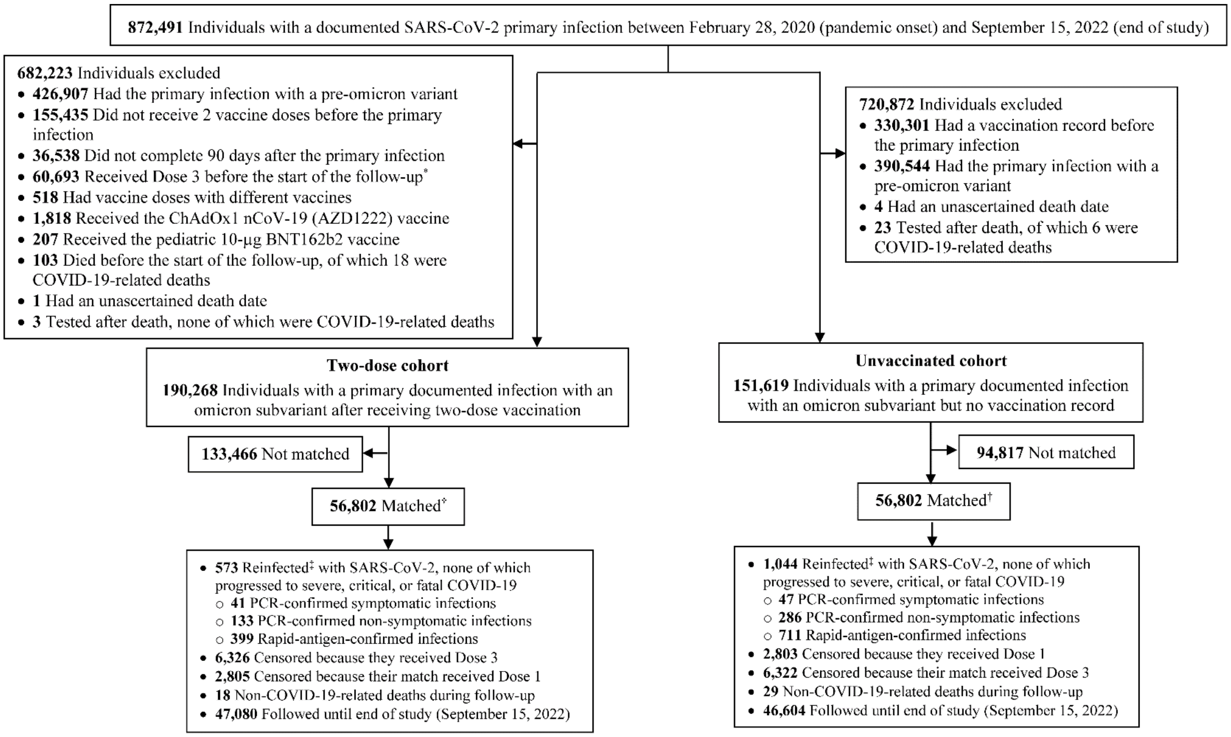

COVID-19 denotes coronavirus disease 2019, PCR polymerase chain reaction, and SARS-CoV-2 severe acute respiratory syndrome coronavirus 2.

*Each matched pair was followed from 90 days after the primary omicron infection of the individual in the two-dose cohort.

†Cohorts were matched exactly one-to-one by sex, age, nationality, number of coexisting conditions, as well as SARS-CoV-2 testing method, reason for SARS-CoV-2 testing, and calendar week of the SARS-CoV-2 test of the primary omicron infection.

‡SARS-CoV-2 reinfection was defined as a documented infection ≥90 days after an earlier infection.

Fig. S1. Flowchart describing the population selection process for investigating immune protection against reinfection among those who had a primary infection with an omicron subvariant after two-dose vaccination compared to protection among those who had a primary infection with an omicron subvariant but were unvaccinated.

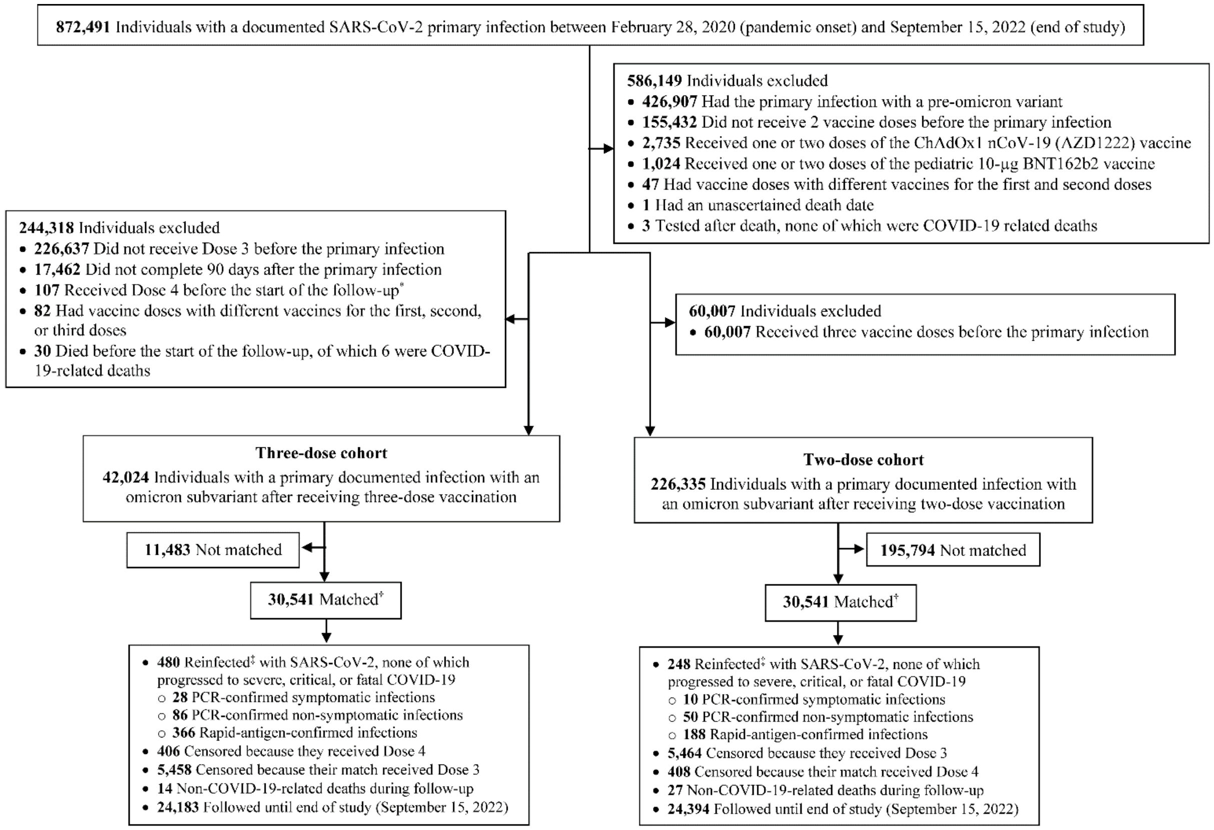

COVID-19 denotes coronavirus disease 2019, PCR polymerase chain reaction, and SARS-CoV-2 severe acute respiratory syndrome coronavirus 2.
*Each matched pair was followed from 90 days after the primary omicron infection of the individual in the three-dose cohort.
†Cohorts were matched exactly one-to-one by sex, age, nationality, number of coexisting conditions, as well as SARS-CoV-2 testing method, reason for SARS-CoV-2 testing, and calendar week of the SARS-CoV-2 test of the primary omicron infection.
‡SARS-CoV-2 reinfection was defined as a documented infection ≥90 days after an earlier infection.

Fig. S2. Flowchart describing the population selection process for investigating immune protection against reinfection among those who had a primary infection with an omicron subvariant after three-dose vaccination compared to protection among those who had a primary infection with an omicron subvariant after two-dose vaccination.

Table S1. Sensitivity analyses. Hazard ratios for incidence of SARS-CoV-2 reinfection in the study investigating immune protection among those who had a primary infection with an omicron subvariant after three-dose vaccination compared to two-dose vaccination.

Epidemiological measure	Cohorts	
**A) Matching by Charlson comorbidity index score[a]**	**Three-dose cohort**	**Two-dose cohort**
Sample size	29,508	29,508
Incident reinfections (n)	478	236
Total follow-up time (person-weeks)	566,587	568,088
Incidence rate of reinfection (per 10,000 person-weeks; 95% CI)	8.4 (7.7 to 9.2)	4.2 (3.7 to 4.7)
Unadjusted hazard ratio for SARS-CoV-2 reinfection (95% CI)	2.04 (1.74 to 2.38)	
Adjusted hazard ratio for SARS-CoV-2 reinfection (95% CI)[b]	1.99 (1.67 to 2.39)	
Hazard ratio for SARS-CoV-2 reinfection additionally adjusted for differences in testing frequency (95% CI)[b]	1.39 (1.16 to 1.67)	
**B) Matching by primary-series vaccine type[c]**	**Three-dose cohort**	**Two-dose cohort**
Sample size	28,357	28,357
Incident reinfections (n)	453	243
Total follow-up time (person-weeks)	546,890	548,008
Incidence rate of reinfection (per 10,000 person-weeks; 95% CI)	8.3 (7.6 to 9.1)	4.4 (3.9 to 5.0)
Unadjusted hazard ratio for SARS-CoV-2 reinfection (95% CI)	1.87 (1.60 to 2.19)	
Adjusted hazard ratio for SARS-CoV-2 reinfection (95% CI)[d]	1.94 (1.62 to 2.33)	
Hazard ratio for SARS-CoV-2 reinfection additionally adjusted for differences in testing frequency (95% CI)[d]	1.43 (1.19 to 1.71)	
**Cohorts who received BNT162b2 primary series**	**Three-dose cohort**	**Two-dose cohort**
Sample size	23,533	23,533
Incident reinfections (n)	405	223
Total follow-up time (person-weeks)	456,085	457,065
Incidence rate of reinfection (per 10,000 person-weeks; 95% CI)	8.9 (8.1 to 9.8)	4.9 (4.3 to 5.6)
Unadjusted hazard ratio for SARS-CoV-2 reinfection (95% CI)	1.82 (1.55 to 2.15)	
Adjusted hazard ratio for SARS-CoV-2 reinfection (95% CI)	1.89 (1.56 to 2.29)	
Hazard ratio for SARS-CoV-2 reinfection additionally adjusted for differences in testing frequency (95% CI)	1.39 (1.15 to 1.68)	
**Cohorts who received mRNA-1273 primary series**		
Sample size	4,804	4,804
Incident reinfections (n)	48	20
Total follow-up time (person-weeks)	90,805	90,943
Incidence rate of reinfection (per 10,000 person-weeks; 95% CI)	5.3 (4.0 to 7.0)	2.2 (1.4 to 3.4)
Unadjusted hazard ratio for SARS-CoV-2 reinfection (95% CI)	2.41 (1.43 to 4.06)	
Adjusted hazard ratio for SARS-CoV-2 reinfection (95% CI)	2.45 (1.37 to 4.39)	
Hazard ratio for SARS-CoV-2 reinfection additionally adjusted for differences in testing frequency (95% CI)	1.83 (1.03 to 3.28)	

CI denotes confidence interval and SARS-CoV-2 severe acute respiratory syndrome coronavirus 2.

[a]Cohorts were matched exactly one-to-one by sex, age, nationality, Charlson comorbidity index, as well as SARS-CoV-2 testing method, reason for SARS-CoV-2 testing, and calendar week of the SARS-CoV-2 test of the primary omicron infection.

[b]Cox regression analysis adjusted for sex, 10-year age groups, 10 nationality groups, Charlson comorbidity index, as well as SARS-CoV-2 testing method, reason for SARS-CoV-2 testing, and calendar week of the SARS-CoV-2 test of the primary omicron infection.

[c]Cohorts were matched exactly one-to-one by sex, age, nationality, number of coexisting conditions, primary-series vaccine type (two doses of BNT162b2 or two doses of mRNA-1273), as well as SARS-CoV-2 testing method, reason for SARS-CoV-2 testing, and calendar week of the SARS-CoV-2 test of the primary omicron infection.

[d]Cox regression analysis adjusted for sex, 10-year age groups, 10 nationality groups, number of coexisting conditions, primary-series vaccine type, as well as SARS-CoV-2 testing method, reason for SARS-CoV-2 testing, and calendar week of the SARS-CoV-2 test of the primary omicron infection.

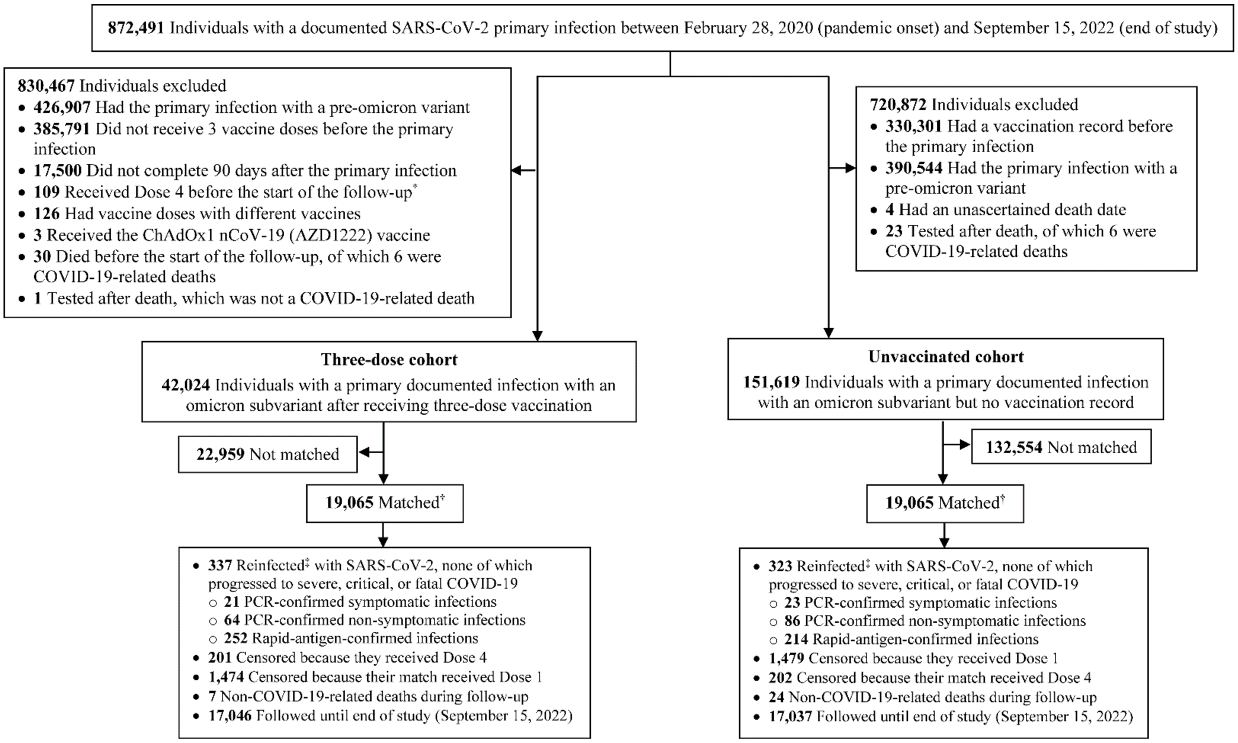

COVID-19 denotes coronavirus disease 2019, PCR polymerase chain reaction, and SARS-CoV-2 severe acute respiratory syndrome coronavirus 2.
*Each matched pair was followed from 90 days after the primary omicron infection of the individual in the three-dose cohort.
†Cohorts were matched exactly one-to-one by sex, age, nationality, number of coexisting conditions, as well as SARS-CoV-2 testing method, reason for SARS-CoV-2 testing, and calendar week of the SARS-CoV-2 test of the primary omicron infection.
‡SARS-CoV-2 reinfection was defined as a documented infection ≥90 days after an earlier infection.

Fig. S3. Flowchart describing the population selection process for investigating immune protection against reinfection among those who had a primary infection with an omicron subvariant after three-dose vaccination compared to protection among those who had a primary infection with an omicron subvariant but were unvaccinated.

Table S2. Baseline characteristics of eligible and matched cohorts in the study investigating immune protection against reinfection among those who had a primary infection with an omicron subvariant after three-dose vaccination compared to those who had a primary infection with an omicron subvariant but were unvaccinated.

Characteristics[a]	Full eligible cohorts			Matched cohorts[b]		
	Three-dose cohort	Unvaccinated cohort	SMD[c]	Three-dose cohort	Unvaccinated cohort	SMD[c]
	N=42,024	N=151,619		N=19,065	N=19,065	
Median age (IQR)—years	40 (34-49)	22 (7-34)	1.39[d]	37 (31-44)	36 (31-44)	0.04[d]
Age—years						
0-9 years	1 (<0.01)	50,360 (33.2)	1.50	1 (0.01)	1 (0.01)	0.00
10-19 years	828 (2.0)	21,980 (14.5)		449 (2.4)	449 (2.4)	
20-29 years	4,234 (10.1)	28,134 (18.6)		3,104 (16.3)	3,104 (16.3)	
30-39 years	14,982 (35.7)	29,247 (19.3)		8,245 (43.3)	8,245 (43.3)	
40-49 years	11,652 (27.7)	13,527 (8.9)		4,675 (24.5)	4,675 (24.5)	
50-59 years	6,680 (15.9)	5,146 (3.4)		1,764 (9.3)	1,764 (9.3)	
60-69 years	2,691 (6.4)	2,127 (1.4)		612 (3.2)	612 (3.2)	
70+ years	956 (2.3)	1,098 (0.7)		215 (1.1)	215 (1.1)	
Sex						
Male	23,930 (56.9)	83,294 (54.9)	0.02	10,354 (54.3)	10,354 (54.3)	0.00
Female	18,094 (43.1)	68,325 (45.1)		8,711 (45.7)	8,711 (45.7)	
Nationality[e]						
Bangladeshi	1,025 (2.4)	2,548 (1.7)	0.48	470 (2.5)	470 (2.5)	0.00
Egyptian	2,547 (6.1)	7,561 (5.0)		763 (4.0)	763 (4.0)	
Filipino	7,835 (18.6)	10,505 (6.9)		3,648 (19.1)	3,648 (19.1)	
Indian	10,734 (25.5)	31,281 (20.6)		5,992 (31.4)	5,992 (31.4)	
Nepalese	696 (1.7)	6,673 (4.4)		540 (2.8)	540 (2.8)	
Pakistani	1,005 (2.4)	6,412 (4.2)		409 (2.2)	409 (2.2)	
Qatari	6,145 (14.6)	37,165 (24.5)		3,591 (18.8)	3,591 (18.8)	
Sri Lankan	781 (1.9)	2,602 (1.7)		363 (1.9)	363 (1.9)	
Sudanese	880 (2.1)	3,690 (2.4)		316 (1.7)	316 (1.7)	
Other nationalities[f]	10,376 (24.7)	43,182 (28.5)		2,973 (15.6)	2,973 (15.6)	
Coexisting conditions						
None	26,945 (64.1)	124,701 (82.3)	0.53	16,026 (84.1)	16,026 (84.1)	0.00
1	6,200 (14.8)	19,358 (12.8)		1,420 (7.5)	1,420 (7.5)	
2	3,751 (8.9)	4,940 (3.3)		619 (3.3)	619 (3.3)	
3+	5,128 (12.2)	2,620 (1.7)		1,000 (5.3)	1,000 (5.3)	
Testing method[g]						
PCR	26,019 (61.9)	91,509 (60.4)	0.03	13,197 (69.2)	13,197 (69.2)	0.00
RA	16,005 (38.1)	60,110 (39.7)		5,868 (30.8)	5,868 (30.8)	
Reason for testing[h]						
Clinical suspicion	7,711 (18.4)	22,817 (15.1)	0.38	3,334 (17.5)	3,334 (17.5)	0.00
Contact tracing	4,432 (10.6)	17,653 (11.6)		1,702 (8.9)	1,702 (8.9)	
Survey	2,604 (6.2)	7,277 (4.8)		1,088 (5.7)	1,088 (5.7)	
Individual request	2,969 (7.1)	9,342 (6.2)		1,212 (6.4)	1,212 (6.4)	
Healthcare routine testing	943 (2.2)	2,426 (1.6)		180 (0.9)	180 (0.9)	
Pre-travel	9,836 (23.4)	24,782 (16.3)		6,299 (33.0)	6,299 (33.0)	
Port of entry	1,883 (4.5)	21,244 (14.0)		653 (3.4)	653 (3.4)	
Other	105 (0.3)	374 (0.3)		3 (0.02)	3 (0.02)	
Not specified	11,541 (27.5)	45,704 (30.1)		4,594 (24.1)	4,594 (24.1)	

IQR denotes interquartile range, PCR, polymerase chain reaction, RA, rapid antigen, SARS-CoV-2 severe acute respiratory syndrome coronavirus 2, and SMD standardized mean difference.

[a]These characteristics are ascertained at the start of follow-up of the study cohorts.

[b]Cohorts were matched exactly one-to-one by sex, age, nationality, number of coexisting conditions, as well as SARS-CoV-2 testing method, reason for SARS-CoV-2 testing, and calendar week of the SARS-CoV-2 test of the primary omicron infection.

[c]SMD is the difference in the mean of a covariate between groups divided by the pooled standard deviation. An SMD ≤0.1 indicates adequate matching.

[d]SMD is for the mean difference between groups divided by the pooled standard deviation.

[e]Nationalities were chosen to represent the most populous groups in Qatar.

[f]These comprise up to 157 other nationalities in the unmatched cohorts, and 73 other nationalities in the matched cohorts.

[g]The testing method that was used to ascertain the omicron infection that made the person eligible for inclusion in the cohort.

[h]The reason for testing of the SARS-CoV-2 test that ascertained the omicron infection that made the person eligible for inclusion in the cohort.

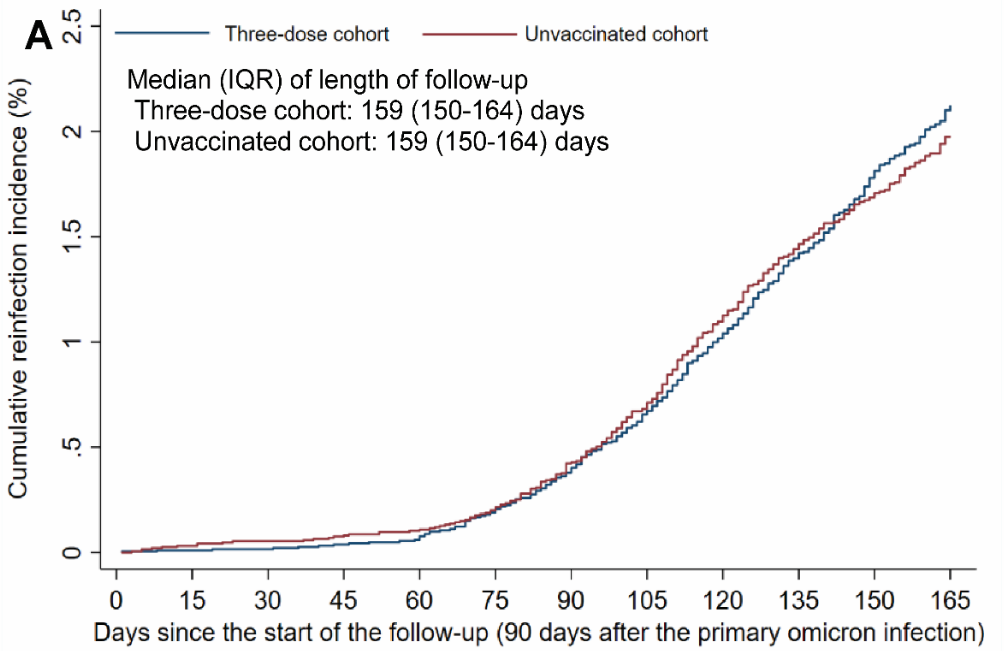

Time (days)	0	15	30	45	60	75	90	105	120	135	150	165
No. at risk												
Three-dose cohort	19,065	18,509	18,216	17,933	17,739	17,559	17,365	17,164	16,823	16,263	14,395	4,576
Unvaccinated cohort	19,065	18,507	18,209	17,924	17,725	17,545	17,346	17,148	16,800	16,242	14,391	4,593

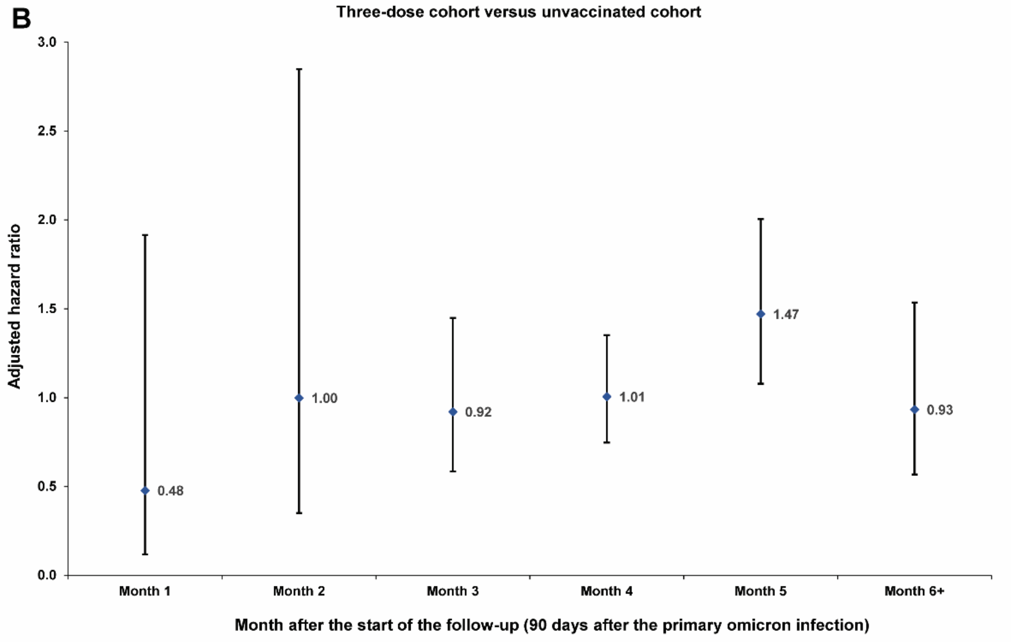

IQR denotes interquartile range

Fig. S4. A) Cumulative incidence of and B) adjusted hazard ratio by month of follow-up for SARS-CoV-2 reinfection among those who had a primary infection with an omicron subvariant after three-dose vaccination compared to those who had a primary infection with an omicron subvariant but were unvaccinated. Error bars indicate confidence intervals.

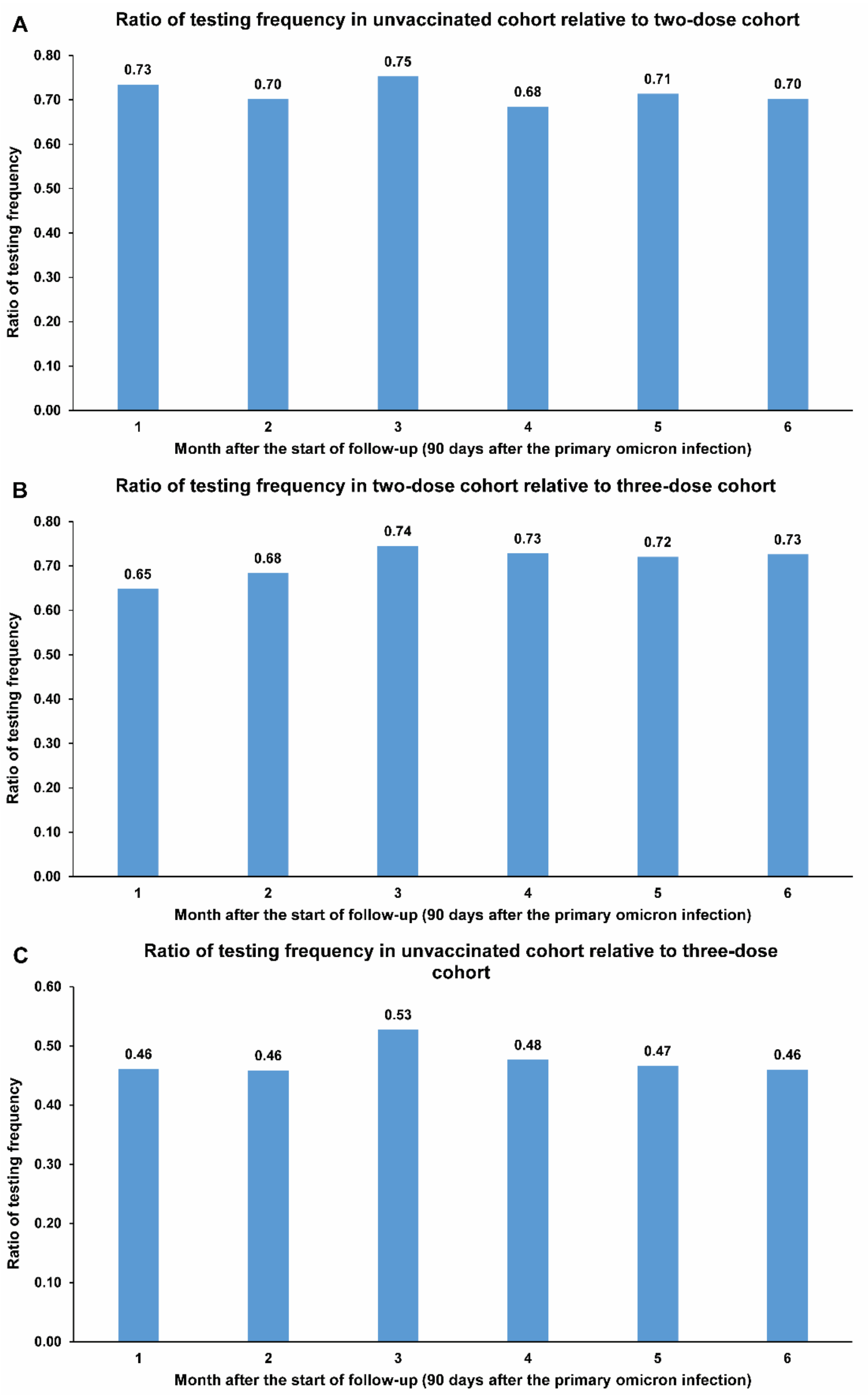

Fig. S5. Ratio of testing frequency in the matched cohorts of studies investigating immune protection among those who had a primary infection with an omicron subvariant, but different vaccination histories.

Table S3. STROBE checklist for cohort studies.

	Item No	Recommendation	Main Text page
**Title and abstract**	1	(*a*) Indicate the study's design with a commonly used term in the title or the abstract<br>(*b*) Provide in the abstract an informative and balanced summary of what was done and what was found	Abstract<br>Abstract
**Introduction**			
Background/rationale	2	Explain the scientific background and rationale for the investigation being reported	Introduction
Objectives	3	State specific objectives, including any prespecified hypotheses	Introduction
**Methods**			
Study design	4	Present key elements of study design early in the paper	Intoduction (paragraph 4) & Materials & Methods ('Study design and cohorts' & 'Cohort matching and follow-up')
Setting	5	Describe the setting, locations, and relevant dates, including periods of recruitment, exposure, follow-up, and data collection	Materials & Methods ('Study population and data sources', 'Study design and cohorts' & 'Cohort matching and follow-up', & Figs. S1-S3
Participants	6	(*a*) Give the eligibility criteria, and the sources and methods of selection of participants. Describe methods of follow-up<br>(*b*) For matched studies, give matching criteria and number of exposed and unexposed	Materials & Methods ('Study design and cohorts' & 'Cohort matching and follow-up', & Figs. S1-S3
Variables	7	Clearly define all outcomes, exposures, predictors, potential confounders, and effect modifiers. Give diagnostic criteria, if applicable	Materials & Methods ('Study design and cohorts' & 'Cohort matching and follow-up', 'Comorbidity classification', 'Laboratory methods', 'COVID-19 severity, criticality, and fatality classification'), Table 1, & Table S2
Data sources/ measurement	8*	For each variable of interest, give sources of data and details of methods of assessment (measurement). Describe comparability of assessment methods if there is more than one group	Materials & Methods ('Study population and data sources', 'Comorbidity classification', 'Laboratory methods', 'COVID-19 severity, criticality, and fatality classification', & 'Statistical analysis', paragraph 1), Table 1, & Table S2
Bias	9	Describe any efforts to address potential sources of bias	Materials & Methods ('Cohort matching and follow-up' & 'Statistical analysis', paragraph 2)
Study size	10	Explain how the study size was arrived at	Figs. S1-S3
Quantitative variables	11	Explain how quantitative variables were handled in the analyses. If applicable, describe which groupings were chosen and why	Materials & Methods ('Cohort matching and follow-up' & 'Statistical analysis', paragraph 2), Table 1, & Table S2
Statistical methods	12	(*a*) Describe all statistical methods, including those used to control for confounding	Materials & Methods ('Statistical analysis')
		(*b*) Describe any methods used to examine subgroups and interactions	Materials & Methods ('Statistical analysis', paragraphs 2-4)
		(*c*) Explain how missing data were addressed	Not applicable, see Materials & Methods ('Study population and data sources')
		(*d*) If applicable, explain how loss to follow-up was addressed	Not applicable, see Materials & Methods ('Study population and data sources')
		(*e*) Describe any sensitivity analyses	Materials & Methods ('Statistical analysis', paragraph 3)
**Results**			
Participants	13*	(a) Report numbers of individuals at each stage of study—eg numbers potentially eligible, examined for eligibility, confirmed eligible, included in the study, completing follow-up, and analysed<br>(b) Give reasons for non-participation at each stage<br>(c) Consider use of a flow diagram	Figs. S1-S3
Descriptive data	14	(a) Give characteristics of study participants (eg demographic, clinical, social) and information on exposures and potential confounders	Results ('Two-dose cohort versus unvaccinated cohort', paragraphs 1 & 2, 'Three-dose cohort versus two-dose cohort', paragraphs 1 & 2, & 'Three-dose cohort versus unvaccinated cohort', paragraph 1), Table 1, & Table S2

		(b) Indicate number of participants with missing data for each variable of interest	Not applicable, see Materials & Methods ('Study population and data sources')
		(c) Summarise follow-up time (eg, average and total amount)	Results ('Two-dose cohort versus unvaccinated cohort', paragraph 2, & 'Three-dose cohort versus two-dose cohort', paragraph 2), Fig. 1, Table 2, & Fig. S4A
Outcome data	15	Report numbers of outcome events or summary measures over time	Results ('Two-dose cohort versus unvaccinated cohort', paragraphs 3 & 4, 'Three-dose cohort versus two-dose cohort', paragraphs 3 & 4, & 'Three-dose cohort versus unvaccinated cohort', paragraph 2), Fig. 1, Table 2, & Fig. S4
Main results	16	(a) Give unadjusted estimates and, if applicable, confounder-adjusted estimates and their precision (eg, 95% confidence interval). Make clear which confounders were adjusted for and why they were included	Results ('Two-dose cohort versus unvaccinated cohort', paragraphs 3 & 4, 'Three-dose cohort versus two-dose cohort', paragraphs 3 & 4, & 'Three-dose cohort versus unvaccinated cohort', paragraph 2), Fig. 1, Table 2, & Fig. S4
		(b) Report category boundaries when continuous variables were categorized	Table 1 & Table S2
		(c) If relevant, consider translating estimates of relative risk into absolute risk for a meaningful time period	Not applicable
Other analyses	17	Report other analyses done—eg analyses of subgroups and interactions, and sensitivity analyses	Results ('Two-dose cohort versus unvaccinated cohort', paragraph 4, 'Three-dose cohort versus two-dose cohort', paragraphs 4-6, & 'Three-dose cohort versus unvaccinated cohort', paragraph 2-3), Fig. 2, Table S1, Fig. S4B, & Fig. S5
Discussion			
Key results	18	Summarise key results with reference to study objectives	Discussion, paragraphs 1-10
Limitations	19	Discuss limitations of the study, taking into account sources of potential bias or imprecision. Discuss both direction and magnitude of any potential bias	Discussion, paragraphs 11-16
Interpretation	20	Give a cautious overall interpretation of results considering objectives, limitations, multiplicity of analyses, results from similar studies, and other relevant evidence	Discussion, paragraph 17
Generalisability	21	Discuss the generalisability (external validity) of the study results	Discussion, paragraphs 11-16
Other information			
Funding	22	Give the source of funding and the role of the funders for the present study and, if applicable, for the original study on which the present article is based	Funding
